# A new order of fishes as hosts of blood flukes (Aporocotylidae); description of a new genus and three new species infecting squirrelfishes (Holocentriformes, Holocentridae) on the Great Barrier Reef

**DOI:** 10.1051/parasite/2021072

**Published:** 2021-11-08

**Authors:** Scott C. Cutmore, Thomas H. Cribb

**Affiliations:** The University of Queensland, School of Biological Sciences Brisbane Queensland 4072 Australia

**Keywords:** Aporocotylidae, Blood fluke, Holocentridae, Squirrelfish, Great Barrier Reef, Australia

## Abstract

A new genus and three new species of blood flukes (Aporocotylidae) are described from squirrelfishes (Holocentridae) from the Great Barrier Reef. *Holocentricola rufus* n. gen., n. sp. is described from *Sargocentron rubrum* (Forsskål), from off Heron Island, southern Great Barrier Reef, and Lizard Island, northern Great Barrier Reef, Australia. *Holocentricola exilis* n. sp. and *Holocentricola coronatus* n. sp. are described from off Lizard Island, *H. exilis* from *Neoniphon sammara* (Forsskål) and *H. coronatus* from *Sargocentron diadema* (Lacepède). Species of the new genus are distinct from those of all other aporocotylid genera in having a retort-shaped cirrus-sac with a distinct thickening at a marginal male genital pore. The new genus is further distinct in the combination of a lanceolate body, X-shaped caeca, posterior caeca that are longer than anterior caeca, a single, post-caecal testis that is not deeply lobed, a post-caecal, post-testis ovary that is not distinctly bi-lobed, and a post-ovarian uterus. The three new species can be morphologically delineated based on the size and row structure of the marginal spines, as well by total length, oesophagus and caecal lengths, and the position of the male genital pore, testes and ovary relative to the posterior extremity. The three species of *Holocentricola* are genetically distinct from each other based on *cox*1 mtDNA and ITS2 rDNA data, and in phylogenetic analyses of 28S rDNA form a well-supported clade sister to species of *Neoparacardicola* Yamaguti, 1970. This is the first report of aporocotylids from fishes of the family Holocentridae and the order Holocentriformes.

## Abstract (fr)

Un nouveau genre et trois nouvelles espèces de digènes du sang (Aporocotylidae) sont décrits de poissons-écureuils (Holocentridae) de la Grande Barrière de Corail. *Holocentricola rufus* n. gen., n. sp. est décrit de *Sargocentron rubrum* (Forsskål), de Heron Island, au sud de la Grande barrière de corail, et de Lizard Island, au nord de la Grande barrière de corail, en Australie. *Holocentricola exilis* n. sp. et *Holocentricola coronatus* n. sp. sont décrits de Lizard Island, *H. exilis* de *Neoniphon sammara* (Forsskål) et *H. coronatus* de *Sargocentron diadema* (Lacepède). Les espèces du nouveau genre se distinguent de celles de tous les autres genres d’Aporocotylidae en ce qu’elles ont un sac du cirre en forme de cornue avec un épaississement distinct au niveau d’un pore génital mâle marginal. Le nouveau genre se distingue en outre par la combinaison d’un corps lancéolé, de caeca en forme de X, de caeca postérieurs plus longs que les caeca antérieurs, d’un seul testicule post-caecal qui n’est pas profondément lobé, d’un ovaire post-caecal et post-testiculaire qui n’est pas distinctement bilobé, et d’un utérus post-ovarien. Les trois nouvelles espèces peuvent être délimitées morphologiquement en fonction de la taille et de la structure des rangées des épines marginales, ainsi que de la longueur totale, de la longueur de l’œsophage et des caeca, et de la position du pore génital mâle, des testicules et de l’ovaire par rapport à l’extrémité postérieure. Les trois espèces d’*Holocentricola* sont génétiquement distinctes les unes des autres sur la base des données d’ADNr *cox1* et d’ADNr ITS2, et forment un groupe-frère bien soutenu des espèces de *Neoparacardicola* Yamaguti, 1970 dans les analyses phylogénétiques de l’ADNr 28S. Il s’agit du premier signalement d’Aporocotylidae chez des poissons de la famille des Holocentridae et de l’ordre des Holocentriformes.

## Introduction

Fishes of Heron and Lizard Islands, on the southern and northern Great Barrier Reef, respectively, have been the focus of extensive blood fluke research over the last two decades; thirty aporocotylid species have been reported from 14 teleost families from these locations. As part of a PhD study, Nolan and Cribb [34, 37, 38] described eight species of *Phthinomita* Nolan & Cribb, 2006 from siganids, mullids and a labrid, six species of *Cardicola* Short, 1953 from siganids and a lutjanid, three species of *Braya* Nolan & Cribb, 2006 from scarids, a species of *Pearsonellum* Overstreet & Køie, 1989 from a serranid, and a species of *Ankistromeces* Nolan & Cribb, 2004 from a siganid. Nolan et al. [39] later described two *Cardicola* species from Lizard Island, one from each a lutjanid and a scombrid, and Nolan et al. [40] described a new species of *Phthinomita* from an apogonid. As part of another PhD study, Yong et al. [70–72] described three species of *Cardicola*, one from each of an apogonid, balistid and chanid, and two species of *Psettarium* Goto & Ozaki, 1930 from tetraodontiforms. Yong and Cribb [67] described a new genus and species from a tetraodontid, and Yong et al. [68] surveyed butterflyfishes (Chaetodontidae) from the Great Barrier Reef, reporting *Elaphrobates chaetodontis* (Yamaguti, 1970) Yong, Cribb & Cutmore, 2021 from 19 chaetodontid species. Recent blood fluke surveys at these locations have led to the re-collection of many of these known species [15], but examination of fish families not previously surveyed is revealing further aporocotylid richness in the region.

Holocentrids, squirrelfishes and soldierfishes, are an important family of nocturnal, semi-cryptic, tropical fishes, commonly found under ledges and in caves on coral reefs. Australia boasts a rich fauna of holocentrids, with 34 species from six genera reported from the region [4]. These fishes have received surprisingly little helminthological attention in Australian waters. Despite being reported as hosts of acanthocolpids, bivesiculids, derogenids, didymozoids, lepocreadiids, opecoelids, and zoogonids elsewhere, there have been just two reports of trematodes infecting Australian holocentrids; *Bivesicula claviformis* Yamaguti, 1934 (Bivesiculidae) and *Lecithochirium cirrhiti* (Manter & Pritchard, 1960) Yamaguti, 1970 (Hemiuridae) were reported from *Sargocentron rubrum* (Forsskål) by Koryakovtseva [22] and Bray et al. [5], respectively.

During recent helminthological examinations of fishes from off Heron and Lizard Islands, blood flukes were collected from three holocentrid species. These specimens represent a genus and three species, new to science, which are formally described and characterised phylogenetically below.

## Materials and methods

### Ethics

Fishes were handled and euthanised following all applicable institutional, national and international guidelines for the care and use of animals. Fishes were collected under Great Barrier Reef Marine Park Authority Permits G16/38038.1 and G19/42323.1, General Fisheries Permits 187264 and 202440, and dissected under Animal Ethics Approval Certificate SBS/454/18.

### Specimen collection

Holocentrid fishes were collected from off Heron Island, southern Great Barrier Reef, and Lizard Island, northern Great Barrier Reef (Queensland, Australia), *via* spearfishing and hand netting. Some gill filaments were removed and examined for the presence of eggs following Yong et al. [68]. Gill arches were removed and placed in saline solution (0.85% NaCl solution). The hemibranchs of each arch were separated, the branchial arteries removed and squeezed or ripped apart. The hemibranchs were then cut into small pieces and washed using the gut-wash approach of Cribb and Bray [9]. The heart was removed, placed in saline solution and each section opened separately. Some of the ventricle tissue was then squashed and examined for the presence of eggs following Yong et al. [68]. The liver was removed, placed in saline and the vessels in the liver mass cut open. The liver was then roughly ripped apart and washed using the gut-wash approach. The head was then cut in half down the midline and washed using the gut-wash approach. The remaining body was then split along the vertebral column and washed using the gut-wash approach. Aporocotylids were washed in vertebrate saline, fixed by pipetting into near-boiling saline, and preserved in 70% ethanol for parallel morphological and molecular characterisation. Some individual worms were processed for both morphological and molecular analysis (hologenophores, *sensu* Pleijel et al. [48]). Species were delineated using an integrative interpretation of morphological, ecological, and genetic data, following the criteria of trematode species recognition proposed by Bray et al. [6] (i.e. reciprocal monophyly in the most discriminating available molecular marker + distinction in morphology or host distribution). Prevalence figures combine any evidence of current infection, i.e. adult worms or fresh eggs lodged in gill tissue.

### Morphological analysis

Specimens for morphological analysis were washed in fresh water, stained in Mayer’s haematoxylin, destained in a solution of 1.0% HCl and neutralised in 1.0% ammonium hydroxide solution. Specimens were then dehydrated through a graded ethanol series, cleared in methyl salicylate and mounted in Canada balsam. Measurements were made using an Olympus SC50 digital camera mounted on an Olympus BX-53 compound microscope using cellSens Standard imaging software. Measurements are in micrometres (μm) and given as a range followed by the mean in parentheses. Where length is followed by breadth, the two measurements are separated by “×”. Drawings were made using an Olympus BX-53 compound microscope and drawing tube.

### Molecular sequencing and phylogenetic analysis

Specimens for molecular analysis were processed according to the protocols used by Cribb et al. [12] and Wee et al. [63]. Sequence data were generated from adult worms (whole or hologenophore specimens) and from eggs lodged in gill filaments. Eggs were not removed from the gill, rather the egg mass and gill filament tip were digested together. Following Blasco-Costa et al. [3], three genetic markers were sequenced, the second internal transcribed spacer region (ITS2 rDNA), the large (28S) ribosomal subunit RNA coding region and the *cox*1 mitochondrial region (*cox*1 mtDNA). The complete ITS2 rDNA region (with flanking 5.8S and 28S regions) was amplified and sequenced using the primers 3S [30] or GA1 [2] and ITS2.2 [10], the partial D1–D3 28S rDNA region using LSU5 [24], 300F [26], ECD2 [25] and 1500R [54] and the partial cox1 region using Dig_cox1Fa [63] and Dig_cox1R [63]. Geneious^®^ version 10.2.6 [21] was used to assemble and edit contiguous sequences.

ITS2 and *cox*1 sequence data generated during this study were aligned in MEGA X [23], with UPGMA clustering for iterations 1 and 2. The *cox*1 alignment was transferred to Mesquite v.3.31 [28], translated (echinoderm/flatworm mitochondrial code) and inspected for internal stop codons. After the correct reading frame was determined, the first column was removed so that the reading frame began on position one, simplifying position-coding in downstream analyses. The final *cox*1 dataset was 474 bp. All codon positions in the *cox*1 dataset were evaluated for substitution saturation, as well as non-stationarity caused by base composition bias. Substitution saturation was assessed using the “Test of substitution saturation by Xia et al.” function [65, 66] as implemented in DAMBE v. 7.2 [64]; no significant substitution saturation was detected. Non-stationarity was assessed using the *χ*^2^ function in PAUP v. 4.0 [57]; significant non-stationarity was not detected. Thus, all codons in the *cox*1 dataset were used in downstream analyses. An unrooted Neighbor-joining analysis was conducted using MEGA X for the *cox*1 dataset to explore species boundaries, with the following parameters “Model/Method = No. of differences”, “Substitutions to Include = *d*: Transitions + Transversions”, “Rates among Sites = Gamma Distributed” and “Gaps/Missing Data Treatment = Pairwise deletion”. Nodal support was estimated by performing 1000 bootstrap replicates. Pairwise differences were estimated for both the ITS2 *cox*1 datasets using the following conditions: “Variance Estimation Method = None”, “Model/Method = No. of differences” and “Substitutions to Include = *d*: Transitions + Transversions” and “Gaps/Missing Data Treatment = Pairwise deletion”.

The partial 28S rDNA sequences generated during this study were aligned with representative sequences of all aporocotylid genera available on GenBank (Table 1). Sequences were aligned using MUSCLE version 3.7 [17] run on the CIPRES portal [29], with ClustalW sequence weighting and UPGMA clustering for iterations 1 and 2. The resultant alignment was refined by eye using Mesquite v.3.31; the ends of the alignment were trimmed, and indels constituting more than three base positions and present in greater than 5% of the sequences in the dataset were removed (leaving a final trimmed dataset of 1254 base positions).

**Table 1:** Collection data and GenBank accession numbers for aporocotylid species incorporated in the 28S analyses.

Species	Host species	GenBank accession #	Reference
Aporocotylidae Odhner, 1905			
*Acipensericola glacialis* Warren & Bullard in Warren, Roberts, Arias, Koenigs & Bullard, 2017	*Acipenser fulvescens* Rafinesque	MF186851	[60]
*Acipensericola petersoni* Bullard, Snyder, Jensen & Overstreet, 2008	*Polyodon spathula* (Walbaum)	KY243879	[44]
*Allocardicola johnpagei* Yong, Cribb & Cutmore, 2021	*Tripodichthys angustifrons* (Hollard)	MZ264862	[73]
*Ankistromeces kawamurai* Cutmore, Yong, Reimer, Shirakashi, Nolan & Cribb, 2021	*Siganus spinus* (Linnaeus)	MZ889038	[15]
*Ankistromeces mariae* Nolan & Cribb, 2004	*Meuschenia freycineti* (Quoy & Gaimard)	MF140288	[7]
*Ankistromeces olsoni* Nolan & Cribb, 2006	*Siganus fuscescens* (Houttuyn)	MF140287	[7]
*Aporocotyle argentinensis* Smith, 1969	*Merluccius hubbsi* Marini	JX094803	[19]
*Aporocotyle mariachristinae* Hernández-Orts, Alama-Bermejo, Carrillo, García, Crespo, Raga & Montero, 2012	*Genypterus blacodes* (Forster)	JX094802	[19]
*Aporocotyle michaudi* Santoro, Cipriani, Pankov & Lawton, 2015	*Trematomus bernacchii* Boulenger	KR025807	[51]
*Aporocotyle spinosicanalis* Williams, 1958	*Merluccius merluccius* (Linnaeus)	AY222177	[43]
*Braya jexi* Nolan & Cribb, 2006	*Scarus frenatus* Lacepède	MZ264863	[73]
*Braya psittacus* Nolan & Cribb, 2006	*Scarus ghobban* Forsskål	MZ264864	[73]
*Braya yantschi* Nolan & Cribb, 2006	*Chlorurus microrhinos* (Bleeker)	MZ264865	[73]
*Cardallagium anthicum* (Bullard & Overstreet, 2006) Yong, Cutmore, Jones, Gauthier & Cribb, 2017	*Rachycentron canadum* (Linnaeus)	KX840316	[59]
*Cardicola abu* Yong, Cutmore & Cribb, 2018	*Abudefduf whitleyi* Allen & Robertson	MH161379	[72]
*Cardicola auratus* Holzer, Montero, Repullés, Sitjà-Bobadilla, Alvarez-Pellitero, Zarza & Raga, 2008	*Sparus aurata* Linnaeus	AM910616	[20]
*Cardicola bullardi* Nolan, Miller, Cutmore, Cantacessi & Cribb, 2014	*Scomberomorus munroi* Collette & Russo	KX523190	[70]
*Cardicola forsteri* Cribb, Daintith & Munday, 2000	*Thunnus orientalis* (Temminck & Schlegel)	KT119353	[53]
*Cardicola langeli* Bullard, 2013	*Archosargus probatocephalus* (Walbaum)	MW158544	[62]
*Cardicola mediterraneus* Palacios-Abella, Montero, Merella, Mele, Raga & Repullés-Albelda, 2021	*Sparus aurata*	MW810092	[47]
*Cardicola opisthorchis* Ogawa, Ishimaru, Shirakashi, Takami & Grabner, 2011	*Terebella* sp.	AB829900	[56]
*Cardicola orientalis* Ogawa, Tanaka, Sugihara & Takami, 2010	*Thunnus orientalis* (Temminck & Schlegel)	HQ324225	[42]
*Cardicola uterohamus* Warren & Bullard in Warren, Bakenhaster, Dutton, Ksepka & Bullard, 2021	*Hyporthodus flavolimbatus* (Poey)	MW147714	[62]
*Chanicola jiigurru* (Yong, Cutmore, Miller, Wee & Cribb, 2016) Yong, Cribb & Cutmore, 2021	*Chanos chanos* (Forsskål)	KX463506	[70]
*Chanicola suni* (Yong, Cutmore, Miller, Wee & Cribb, 2016) Yong, Cribb & Cutmore, 2021	*Chanos chanos* (Forsskål)	KX463511	[70]
*Chimaerohemecus trondheimensis* van der Land, 1967	*Chimaera monstrosa* Linnaeus	AY157239	[27]
*Elaphrobates beveridgei* (Nolan, Miller, Cutmore, Cantacessi & Cribb, 2014) Yong, Cribb & Cutmore, 2021	*Lutjanus argentimaculatus* (Forsskål)	KX523188	[70]
*Elaphrobates chaetodontis* (Yamaguti, 1970) Yong, Cribb & Cutmore, 2021	*Chaetodon rainfordi* McCulloch	KX523192	[70]
*Elaphrobates milleri* (Nolan & Cribb, 2006) Yong, Cribb & Cutmore, 2021	*Lutjanus bohar* (Forsskål)	MZ264867	[73]
*Electrovermis zappum* Warren & Bullard, 2019	*Narcine bancroftii* (Griffith & Smith)	MN244242	[58]
*Elopicola bristowi* Orélis-Ribeiro & Bullard in Orélis-Ribeiro, Halanych, Dang, Bakenhaster, Arias & Bullard, 2017	*Elops hawaiensis* Regan	KY243881	[44]
*Elopicola franksi* Orélis-Ribeiro & Bullard in Orélis-Ribeiro, Halanych, Dang, Bakenhaster, Arias & Bullard, 2017	*Megalops atlanticus* Valenciennes	KY243882	[44]
*Elopicola nolancribbi* Bullard, 2014	*Elops saurus* Linnaeus	KY243880	[44]
*Gymnurahemecus bulbosus* Warren, Ruiz, Whelan, Kritsky & Bullard, 2019	*Gymnura micrura* (Bloch & Schneider)	MH555433	[61]
*Littorellicola billhawkinsi* Bullard, 2010	*Trachinotus carolinus* (Linnaeus)	MW152328	[62]
*Neoparacardicola nasonis* Yamaguti, 1970	*Naso unicornis* (Forsskål)	AY222179	[43]
*Neoparacardicola* cf. *nasonis*	*Naso unicornis*	MF140278	[7]
*Ogawaia glaucostegi* Cutmore, Cribb & Yong, 2018	*Glaucostegus typus* (Anonymous [Bennett])	MF503308	[14]
*Paradeontacylix godfreyi* Hutson & Whittington, 2006	*Seriola lalandi* Valenciennes	AM489597	[49]
*Paradeontacylix grandispinus* Ogawa & Egusa, 1986	*Seriola dumerili* (Risso)	AM489596	[49]
*Paradeontacylix iberica* Repullés-Albelda, Montero, Holzer, Ogawa, Hutson & Raga, 2008	*Seriola dumerili*	AM489593	[49]
*Phthinomita abdita* Cutmore, Yong, Reimer, Shirakashi, Nolan & Cribb, 2021	*Choerodon cephalotes* (Castelnau)	MZ889041	[15]
*Phthinomita jonesi* Nolan & Cribb, 2006	*Siganus lineatus* (Valenciennes)	MF140277	[7]
*Phthinomita poulini* Nolan & Cribb, 2006	*Parupeneus barberinus* (Lacepède)	MF140275	[7]
*Plethorchis acanthus* Martin, 1975	*Mugil cephalus* Linnaeus	AY222178	[7]
*Psettarium ogawai* Yong, Cutmore, Bray, Miller, Semarariana, Palm & Cribb, 2016	*Arothron reticularis* (Bloch & Schneider)	KX284694	[69]
*Psettarium pandora* Yong, Cutmore, Jones, Gauthier & Cribb, 2018	*Ostracion cubicum* Linnaeus	MG709046	[71]
*Psettarium pulchellum* Yong, Cutmore, Bray, Miller, Semarariana, Palm & Cribb, 2016	*Arothron manilensis* (Marion de Procé)	MG709049	[71]
*Rhaphidotrema kiatkiongi* Yong & Cribb, 2011	*Arothron hispidus* (Linnaeus)	MZ264868	[73]
*Skoulekia bogaraveo* Palacios-Abella, Raga, Mele & Montero, 2018	*Pagellus bogaraveo* (Brünnich)	MF959771	[46]
*Skoulekia erythrini* Palacios-Abella, Georgieva, Mele, Raga, Isbert, Kostadinova & Montero, 2017	*Pagellus erythrinus* (Linnaeus)	MF043944	[45]
*Skoulekia meningialis* Alama-Bermejo, Montero, Raga & Holzer, 2011	*Diplodus vulgaris* (Geoffroy Saint-Hilaire)	FN652293	[1]
*Spirocaecum covacinae* (Nolan & Cribb, 2006) Yong, Cribb & Cutmore, 2021	*Siganus punctatus* (Schneider & Forster)	MF140283	[7]
*Spirocaecum lafii* (Nolan & Cribb, 2006) Yong, Cribb & Cutmore, 2021	*Siganus fuscescens* (Houttuyn)	MF140282	[7]
*Spirocaecum mogilae* (Brooks, Cribb, Yong & Cutmore, 2017) Yong, Cribb & Cutmore, 2021	*Siganus fuscescens*	MF140281	[7]
*Aporocotylidae* sp. NSW1	*Plebidonax deltoides* (Lamarck)	MF503307	[13]

Bayesian inference and maximum likelihood analyses of the 28S dataset were conducted to explore relationships among these taxa. Bayesian inference analysis was performed using MrBayes version 3.2.7 [50] and maximum likelihood analysis using RAxML version 8.2.12 [55], both run on the CIPRES portal. The best nucleotide substitution model was estimated using jModelTest version 2.1.10 [16]. Both the Akaike Information Criterion (AIC) and Bayesian Information Criterion (BIC) predicted the TPM3uf + *I* + Γ model as the best estimator; Bayesian inference and maximum likelihood analyses were conducted using the closest approximation to this model. Nodal support in the maximum likelihood analysis was estimated by performing 1000 bootstrap pseudoreplicates. Bayesian inference analysis was run over 10,000,000 generations (ngen = 10,000,000) with two runs each containing four simultaneous Markov Chain Monte Carlo (MCMC) chains (nchains = 4) and every 1000th tree saved. Bayesian inference analysis used the following parameters: nst = 6, rates = invgamma, ngammacat = 4, and the priors parameters of the combined dataset were set to ratepr = variable. Samples of substitution model parameters, and tree and branch lengths were summarised using the parameters sump burnin = 3000 and sumt burnin = 3000. Aporocotylids of chondrichthyans were designated as functional outgroup taxa, following Warren et al. [61].

## Results

### General results

**Figure 1:**
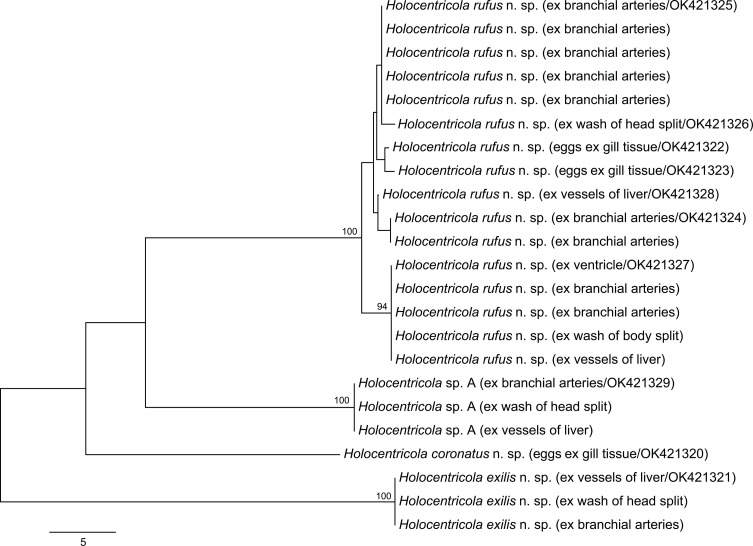
Phylogram from the unrooted Neighbour-joining analysis of the *cox*1 mtDNA dataset. Bootstrap support values are shown at the nodes, with values of <85 not shown. The scale-bar indicates the number of base differences.

Six species of Holocentridae were examined for aporocotylids on the Great Barrier Reef: 30 *Neoniphon sammara* (Forsskål), 10 *Myripristis murdjan* (Forsskål), nine *S. diadema* (Lacepède), nine *S. spiniferum* (Forsskål), three *Sargocentron caudimaculatum* (Rüppell), and two *S. rubrum* from off Lizard Island; and 17 *S. rubrum* and one *N. sammara* from off Heron Island. Adult aporocotylids were collected from *N. sammara* and *S. diadema* off Lizard Island and from *S. rubrum* off Heron Island. Adult worms were found in the branchial arteries, heart, vessels of the liver, head split wash and body split wash, but only in one or two of these sites in any individual fish. Eggs lodged in the gill tissue were found in all hosts infected by adult worms, as well as in four *S. rubrum*, three *N. sammara* and one *S. diadema* from which no adults were found; eggs were always concentrated in clusters at the tips of small numbers of gill filaments. Eggs lodged in heart tissue were found in just a single *S. rubrum* which was also infected by adult worms. Sequence data were generated for all host species/infection location combinations, from adults and from eggs. Four *cox*1 genotypes are present (Fig. 1), with one from only *N. sammara* at Lizard Island, one from only *S. diadema* at Lizard Island, one from *S. rubrum* at Heron and Lizard Islands, and one from *S. rubrum* at only Heron Island. Based on genetic, morphometric, and ecological data, we recognize the four genotypes as representing four distinct species belonging to a new genus; three are formally described, with the fourth lacking suitable morphological material.

Family Aporocotylidae Odhner, 1912

### Genus *Holocentricola* n. gen.

urn:lsid:zoobank.org:act:5A09F566-8340-4E4D-8EBA-BA767D613365

#### Diagnosis

Body lanceolate, ventrally concave, broadest at level of testis or caeca, with distinct terminal notch at posterior end, and distinct bulge sometimes present at level of uterus. Tegumental spines arranged in ventro-marginal transverse rows for entire body length, straight for most of body length, those in final 5–10 rows slightly curved with small hook on tip. Rosethorn-shaped or fused spines absent. Oral sucker poorly delineated, weakly muscularised, bearing concentric rows of fine spines. Mouth ventrally subterminal. Oesophagus almost straight to gently sinuous, thick-walled. Caeca form X-shape; intestinal bifurcation in middle third of body. Anterior caeca equal to subequal in length, much shorter than posterior caeca. Posterior caeca equal to subequal in length. Testis single, roughly rectangular, with margins irregularly lobed, immediately posterior to posterior margin of posterior caeca, usually extends laterally beyond lateral nerve cords. External seminal vesicle absent. Vas deferens sometimes widening posteriorly. Cirrus-sac retort-shaped, rounded anteriorly, dramatically narrowed posteriorly; anterior rounded portion contains seminal vesicle and pars prostatica; posterior narrow portion notably thickened at marginal genital pore, contains ejaculatory duct. Seminal vesicle round to ovoid, restricted to anterior, rounded portion of cirrus-sac, joining coiled pars prostatica. Ejaculatory duct long. Male genital pore on sinistral margin at distinct to indistinct marginal notch. Ovary oblong, roughly rectangular or wedge-shaped, medial, with margins irregularly lobed, immediately posterior to testis, usually extending laterally beyond lateral nerve cords. Oviducal seminal receptacle present. Oötype posterior to rest of genitalia, medial to submedial. Uterus weakly convoluted, passing anteriorly between oviduct and dextral side of cirrus-sac, ventrally overlapping posterior portion of ovary, then passing posteriorly, sinistral to cirrus-sac, to female genital pore; distal portion of uterus often forming prominent egg reservoir, creating distinct marginal bulge. Female genital pore dorsal, sinistro-submedial, separate from and anterior to male pore. Eggs *in utero* ovoid to subspherical, very thin-shelled, anoperculate. Vitellarium follicular, distributed from just posterior to dorsal nerve commissure to posterior half of testis or level of ovary, laterally exceeding nerve cords, largely confluent anterior to testis. Excretory vesicle small, saccular. Excretory pore at apex of terminal notch. In circulatory system of holocentrid fishes.

*Type species*: *Holocentricola rufus* n. sp.

*Other species*: *Holocentricola exilis* n. sp.; *Holocentricola coronatus* n. sp.

*Etymology*: This genus is named for the order of fishes it infects (the Holocentriformes) and the Latin -*cola* (dweller or inhabitant). It should be treated as masculine.

### *Holocentricola rufus* n. sp. (Figs. 2A, 3A)

urn:lsid:zoobank.org:act:89CB178C-58C2-431D-AC9A-B4AC0B96C3A7

**Figure 2:**
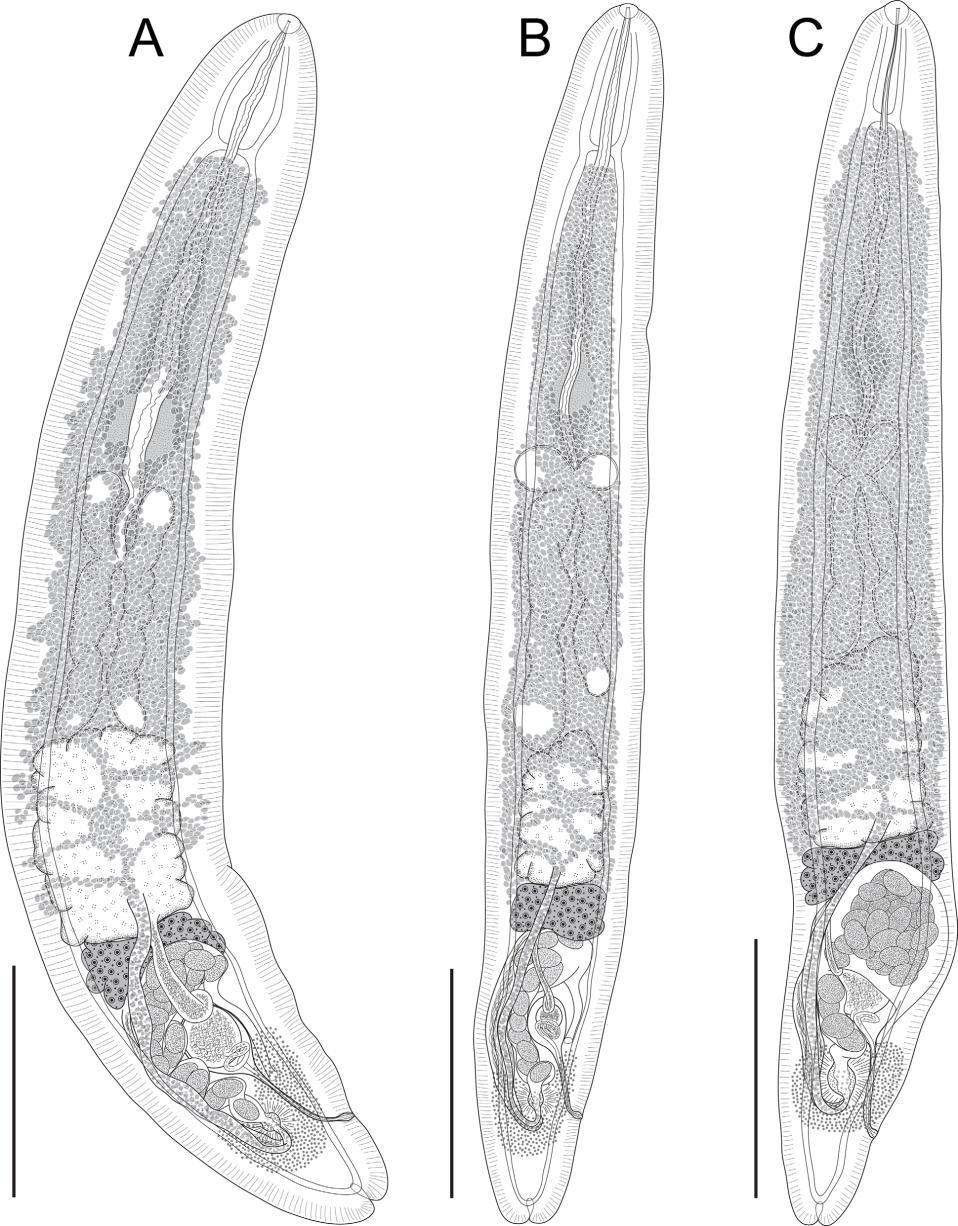
Species of *Holocentricola* from Great Barrier Reef Holocentridae, whole worms, ventral views. (A) *Holocentricola rufus* n. sp. ex *Sargocentron rubrum* from off Heron Island (holotype, QM G239429); (B) *Holocentricola exilis* n. sp. ex *Neoniphon sammara* from off Lizard Island (paratype, QM G239111); (C) *Holocentricola coronatus* n. sp. ex *Sargocentron diadema* from off Lizard Island (holotype, QM G239125). Scale-bars: A–C, 200 μm.

**Figure 3:**
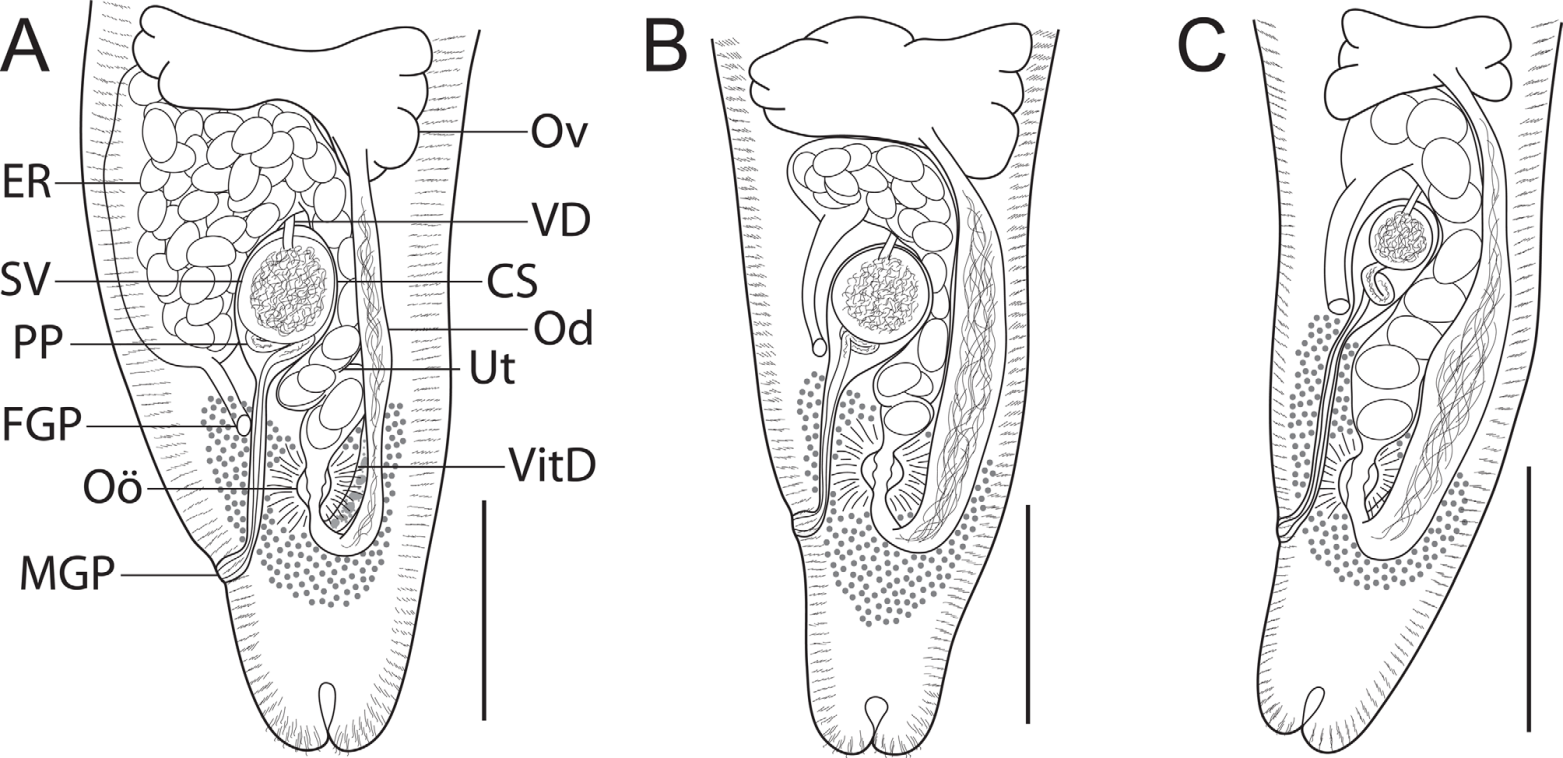
Species of *Holocentricola* from Great Barrier Reef Holocentridae, terminal genitalia, dorsal views; spines illustrated are ventral. (A) *Holocentricola rufus* n. sp. ex *Sargocentron rubrum* from off Heron Island (paratype, QM G239440); (B) *Holocentricola exilis* n. sp. ex *Neoniphon sammara* from off Lizard Island (paratype, QM G239119); (C) *Holocentricola coronatus* n. sp. ex *Sargocentron diadema* from off Lizard Island (paratype, QM G239126). *Abbreviations*: CS, cirrus-sac; ER, egg reservoir; FGP, female genital pore; MGP, male genital pore; Od, oviduct; Oö, oötype; Ov, ovary; PP, pars prostatica; SV, seminal vesicle; Ut, uterus; VD, vas deferens; VitD, vitelline duct. Scale-bars: A–C, 100 μm.

*Type host*: *Sargocentron rubrum* (Forsskål), Red squirrelfish (Holocentriformes: Holocentridae).

*Type locality*: Off Heron Island (23° 27′ S, 151° 55′ E), southern Great Barrier Reef, Australia.

*Other locality*: Off Lizard Island (14° 40′ S, 145° 27′ E), northern Great Barrier Reef, Australia.

*Site in host*: Ventricle, branchial arteries, vessels of liver, wash of head split, wash of body split.

*Prevalence*: 14 of 17 Heron Island (adult worms in 11); 1 of 2 Lizard Island (no adult worms).

*Intensity*: 1–12 worms per fish (mean 4.27), when adult worms were detected.

*Type material*: Holotype (QM G239429) and 24 paratypes (QM G239430–239453), including nine hologenophores.

*Representative DNA sequences*: Partial *cox*1 mtDNA, 16 sequences (seven submitted to GenBank, OK421322–OK421328); ITS2 rDNA, three identical sequences (two submitted to GenBank, OK422500–OK421301); partial 28S rDNA, one sequence (submitted to GenBank, OK422504).

*Etymology*: This species is named from the Latin *rufus* (red) for the type and only host, the Red squirrelfish.

*Description* [based on 25 specimens, including nine hologenophores]: Body lanceolate, ventrally concave, broadest at level of testis or caeca, 976–1290 × 142–222 (1093 × 182), 4.4–7.6 (6.1) times longer than wide; notch usually noticeable at level of male genital pore, sometimes indistinct; distinct terminal notch at posterior end; distinct bulge sometimes present at level of uterus. Tegumental spines arranged in ventro-marginal transverse rows for entire body length, 7–8 long, straight for most of body length, those in final 5–10 rows slightly curved with small hook on tip, 10–11 long. First spine row with 3–4 spines, increasing in number prior to dorsal nerve commissure, 8–9 spines per row for most of body length, decreasing in number posterior to testis, 3 spines in final row; spine rows 14–21 (18) wide in anterior third of body, 17–22 (19) wide in middle third of body, and 11–28 (14) wide in posterior third of body; rows spaced 4 apart. Dorsal nerve commissure 34–54 (46) across, 87–134 (112) from anterior extremity. Nerve cords well-defined, 6–9 (7) in diameter, run length of body, 30–52 (41) from body margin at midbody. Oral sucker poorly delineated, weakly muscularised, 14–28 × 15–22 (16 × 19), bearing concentric rows of fine spines in some specimens, number of rows undetermined. Mouth a simple pore, ventrally subterminal, 6–9 (7) from anterior extremity. Oesophagus almost straight to gently sinuous, thick-walled, 433–597 (480) long. Oesophageal glands enveloping oesophagus posterior to dorsal nerve commissure, thickening and forming distinct glandular bulb immediately anterior to anterior caeca. Caeca form X-shape; intestinal bifurcation in middle third of body, 435–602 (480), or 39.5–48.2% of total body length, from anterior extremity. Anterior caeca equal to subequal in length, shorter than posterior caeca; left anterior caecum 64–94 (77); right anterior caecum 65–100 (79); longer anterior caecum occupying 6.1–8.8% of total body length. Posterior caeca equal to subequal in length, 1.3–2.9 (2.1) times longer than anterior; left posterior caecum 111–222 (157); right posterior caecum 123–213 (162); longer posterior caecum occupying 9.5–19.5% of total body length. Total caecal length 204–308 (245), occupying 16.0–27.1% of body length.

Testis single, roughly rectangular, with margins irregularly lobed, immediately posterior to posterior ends of posterior caeca, extends laterally beyond lateral nerve cords and posteriorly to anterior margin of ovary, 100–233 × 77–154 (171 × 120), occupying 10.1–18.9% of total body length; post-testicular space 250–351 (296), or 25.2–29.2% of body length. Vas deferens originates medially from posterior margin of testis, passing ovary and uterus ventrally, widening posteriorly in some specimens, entering cirrus-sac dorso-anteriorly. External seminal vesicle absent. Cirrus-sac retort-shaped, rounded anteriorly, dramatically narrowed posteriorly; anterior rounded portion 46–79 × 30–55 (62 × 43), contains seminal vesicle and pars prostatica; posterior narrow portion 81–108 (92) long, notably thickened at marginal genital pore, contains ejaculatory duct (un-everted cirrus; everted cirrus not observed), 5–9 (6) wide at midpoint, 8–12 (10) wide at marginal thickening. Seminal vesicle round to ovoid, 25–69 × 26–53 (43 × 39), restricted to anterior, rounded portion of cirrus-sac, joining coiled pars prostatica; prostatic cells not observed. Ejaculatory duct long. Male genital pore on sinistral margin at distinct to indistinct marginal notch, 66–96 (79), or 6.5–8.3% of body length, from posterior extremity.

Ovary wedge-shaped to oblong, medial, with margins irregularly lobed, immediately posterior to testis, sometimes extending laterally beyond lateral nerve cords, 38–74 × 84–154 (58 × 119), 216–301 (249), or 21.4–24.3% of total body length, from posterior extremity. Oviduct originates from posterior margin of ovary, passes posteriorly dorsal to vitelline duct, dextro-lateral and sometimes partially dorsal to ascending portion of uterus, posteriorly curving sinistrally to meet oötype, usually filled with sperm. Oötype posterior to rest of genitalia, medial to submedial, surrounded by Mehlis’ gland, 81–138 (102) from posterior extremity. Uterus weakly convoluted, passing anteriorly between oviduct and dextral side of cirrus-sac, ventrally overlapping posterior portion of ovary, then passing posteriorly, sinistral to cirrus-sac, to female genital pore; distal portion of uterus often forming egg reservoir, creating distinct marginal bulge. Female genital pore dorsal, sinistro-submedial, separate from and anterior to male pore, just posterior to level of constriction dividing anterior and posterior portions of cirrus-sac, 33–51 (39) from sinistral margin, 104–163 (133) from posterior extremity. Eggs *in utero* ovoid to subspherical, very thin-shelled, anoperculate, 22–29 × 12–24 (25 × 17). Vitellarium follicular, distributed from just posterior to dorsal nerve commissure to posterior half of testis, laterally exceeding nerve cords, largely confluent anterior to testes, sometimes interrupted by ends of caeca and oesophageal gland, interrupted partially by testis ventrally and dorsally. Vitelline duct passes ovary ventrally, passing posterio-dextrally to oötype, ventrally overlaps oviduct and sometimes part of ascending portion of uterus, posteriorly curving sinistrally to meet oötype.

Excretory vesicle small, saccular; paired collecting ducts not traceable. Excretory pore at apex of terminal notch.

#### Remarks

*Holocentricola rufus* was found in all body sites examined, with adult worms in the heart (specifically the ventricle), branchial arteries of the gills, the major vessels of the liver, as well as in the wash of head split (gills already removed), and wash of entire body split (head and gills removed); however, specimens of this species were most commonly found infecting the branchial arteries. *cox*1 sequence data were generated for samples from all five infections sites and from eggs lodged in the tips of gill filaments; all sequences form a strongly supported clade in the neighbor-joining analysis, with no division by infection location. No adults were recovered from the single infection from Lizard Island but an ITS2 sequence was generated from eggs lodged in gill tissue; this sequence is identical to those from adult samples from Heron Island.

### *Holocentricola exilis* n. sp. (Figs. 2B, 3B)

urn:lsid:zoobank.org:act:5DAB8EDD-EF49-45DC-95ED-F9437F91B4F0

*Type host*: *Neoniphon sammara* (Forsskål), Slender squirrelfish (Holocentriformes: Holocentridae).

*Type locality*: Off Lizard Island (14° 40′ S, 145° 27′ E), northern Great Barrier Reef, Australia.

*Site in host*: Heart, branchial arteries, vessels of liver, wash of head split.

*Prevalence*: 16 of 30 Lizard Island (adult worms in 13); 0 of 1 Heron Island.

*Intensity*: 1–4 worms per fish (mean 1.53), when adult worms were detected.

*Type material*: Holotype (QM G239110) and 14 paratypes (QM G239111–24).

*Representative DNA sequences*: Partial *cox*1 mtDNA, three identical sequences (one submitted to GenBank, OK421321); ITS2 rDNA, one sequence (submitted to GenBank, OK422499); partial 28S rDNA, one sequence (submitted to GenBank, OK422503).

*Etymology*: This species is named from the Latin *exilis* (slender or thin) for the type and only host, the Slender squirrelfish.

*Description* [based on 15 specimens]: Body lanceolate, ventrally concave, broadest at level of testis or caeca, 961–1232 × 123–190 (1055 × 152), 5.8–7.8 (7.0) times longer than wide; notch usually noticeable at level of male genital pore, sometimes indistinct; distinct terminal notch at posterior end; distinct bulge sometimes present at level of uterus. Tegumental spines arranged in ventro-marginal transverse rows for entire body length, 8–9 long, straight for most of body length, those in final 5–10 rows slightly curved with small hook on tip, 10–11 long. First spine row with 3–4 spines, increasing in number prior to dorsal nerve commissure, 7 spines per row for most of body length, decreasing in number in posterior third of body, 3 spines in final row; spine rows 10–14 (12) wide, spaced 3–4 apart. Dorsal nerve commissure 33–53 (40) across, 89–126 (111) from anterior extremity. Nerve cords well-defined, 6–9 (7) in diameter, run length of body, 24–38 (29) from body margin at midbody. Oral sucker poorly delineated, weakly muscularised, 16–19 × 19–25 (17 × 22), bearing 5 concentric rows of fine spines. Mouth a simple pore, ventrally subterminal, 4–11 (8) from anterior extremity. Oesophagus almost straight to gently sinuous, thick-walled, 359–420 (396) long. Oesophageal glands enveloping oesophagus posterior to dorsal nerve commissure, thickening and forming distinct glandular bulb immediately anterior to anterior caeca. Caeca form X-shape; intestinal bifurcation in middle third of body, 356–424 (397), or 34.4–41.1% of total body length, from anterior extremity. Anterior caeca equal to subequal in length, much shorter than posterior caeca; left anterior caecum 31–57 (40); right anterior caecum 23–64 (43); longer anterior caecum occupying 3.3–6.2% of total body length. Posterior caeca equal to subequal in length, 3.4–8.5 (5.3) times longer than anterior; left posterior caecum 189–259 (210); right posterior caecum 157–281 (217); longer posterior caecum occupying 19.3–24.5% of total body length. Total caecal length 224–295 (260), occupying 22.3–28.6% of body length.

Testis single, roughly rectangular, with margins irregularly lobed, immediately posterior to posterior ends of posterior caeca, usually extends laterally beyond lateral nerve cords and posteriorly to anterior margin of ovary, 98–209 × 61–150 (140 × 103), occupying 10.1–17.0% of total body length; post-testicular space 258–367 (299), or 25.7–30.1% of body length. Vas deferens originates medially from posterior margin of testis, passing ovary and uterus ventrally, widening posteriorly in some specimens, entering cirrus-sac dorso-anteriorly. External seminal vesicle absent. Cirrus-sac retort-shaped, rounded anteriorly, dramatically narrowed posteriorly; anterior rounded portion 45–66 × 29–58 (56 × 42), contains seminal vesicle and pars prostatica; posterior narrow portion 65–93 (73) long, notably thickened at marginal genital pore, contains ejaculatory duct (un-everted cirrus; everted cirrus not observed), 4–7 (5) wide at midpoint, 6–10 (8) wide at marginal thickening. Seminal vesicle roughly round, 25–46 × 23–49 (35 × 35), restricted to anterior, rounded portion of cirrus-sac, joining coiled pars prostatica; prostatic cells not observed. Ejaculatory duct long. Male genital pore on sinistral margin at distinct to indistinct marginal notch, 89–124 (101), or 8.9–10.6% of body length, from posterior extremity.

Ovary oblong to roughly rectangular, medial, with margins irregularly lobed, immediately posterior to testis, usually extending laterally beyond lateral nerve cords, 43–73 × 65–138 (53 × 103); post-ovarian space 216–306 (249), or 21.1–25.4% of total body length. Oviduct originates from posterior margin of ovary, passes posteriorly dorso-lateral to vitelline duct and dextro-lateral to ascending portion of uterus, posteriorly curving sinistrally to meet oötype, usually heavily distended with sperm. Oötype posterior to rest of genitalia, medial, surrounded by Mehlis’ gland, 94–129 (109) from posterior extremity. Uterus weakly convoluted, passing anteriorly between oviduct and dextral side of cirrus-sac, ventrally overlapping posterior portion of ovary, then passing posteriorly, sinistral to cirrus-sac, to female genital pore; distal portion of uterus often forming egg reservoir, creating distinct marginal bulge. Female genital pore dorsal, sinistro-submedial, separate from and anterior to male pore, at level of constriction dividing anterior and posterior portions of cirrus-sac, 16–37 (27) from sinistral margin, 145–205 (167) from posterior extremity. Eggs *in utero* ovoid to subspherical, very thin-shelled, anoperculate, 19–34 × 12–19 (24 × 16). Vitellarium follicular, distributed from just posterior to dorsal nerve commissure to posterior half of testis, rarely to level of ovary, laterally exceeding nerve cords, largely confluent anterior to testis, sometimes interrupted partially by ends of caeca and oesophageal gland, interrupted partially by testis ventrally, completely or partially interrupted by testis dorsally. Vitelline duct passes ovary ventrally, passing posterio-dextrally to oötype, ventrally overlaps oviduct, posteriorly curving sinistrally to meet oötype.

Excretory vesicle small, saccular; paired collecting ducts not traceable. Excretory pore at apex of terminal notch.

#### Remarks

*Holocentricola exilis* was found in four of the five body sites examined (heart, branchial arteries, vessels of liver, wash of head split), but was most commonly found in the wash of the head split and the branchial arteries. The intensity of infection of *H. exilis* was notably lower than that found for *H. rufus* (1–4 worms per fish, mean 1.53 *vs* 1–12 worms per fish, mean 4.27).

### *Holocentricola coronatus* n. sp. (Figs. 2C, 3C)

urn:lsid:zoobank.org:act:4F33844D-83D9-4803-9311-19CFE68CDC5B

*Type host*: *Sargocentron diadema* (Lacepède), Crown squirrelfish (Holocentriformes: Holocentridae).

*Type locality*: Off Lizard Island (14° 40′ S, 145° 27′ E), northern Great Barrier Reef, Australia.

*Site in host*: Ventricle, branchial arteries, vessels of liver, wash of head split.

*Prevalence*: 2 of 9 (adults in one).

*Intensity*: 4 worms in single fish from which adult worms were recovered.

*Type material*: Holotype (QM G239125) and 3 paratypes (QM G239126–28).

*Representative DNA sequences*: Partial *cox*1 mtDNA, one sequence (submitted to GenBank, OK421320); ITS2 rDNA, one sequence (submitted to GenBank, OK422498).

*Etymology*: This species is named from the Latin *coronatus* (crowned) for the type and only host, the Crown squirrelfish.

*Description* [based on four specimens]: Body lanceolate, ventrally concave, broadest at level of testis or caeca, 839–937 × 111–141 (890 × 124), 6.16–8.44 (7.25) times longer than wide; subtle notch at level of male genital pore; distinct terminal notch at posterior end; distinct bulge sometimes present at level of uterus. Tegumental spines arranged in ventro-marginal transverse rows for entire body length, 6 long, straight for most of body length, those in final 5–10 rows slightly curved with small hook on tip, 8–9 long. First spine row with 4 spines, increasing in number after first few rows, 5–6 spines per row for most of body length, decreasing in number posterior to ovary, 3 spines in final row; spine rows 8–9 wide, spaced 3 apart in anterior third of body, 4–5 apart in middle and posterior thirds of body. Dorsal nerve commissure 25–37 (30) across, 80–90 (86) from anterior extremity. Nerve cords well-defined, 5–8 (7) in diameter, run length of body, 22–30 (25) from body margin at midbody. Oral sucker poorly delineated, weakly muscularised, 14–18 × 16–23 (16 × 19), bearing concentric rows of fine spines, number of rows undetermined. Mouth a simple pore, ventrally subterminal, 5–8 (7) from anterior extremity. Oesophagus gently sinuous, thick-walled, 311–349 (337) long. Oesophageal glands enveloping oesophagus posterior to dorsal nerve commissure, thickening and forming glandular bulb distinctly anterior to anterior caeca. Caeca form X-shape; intestinal bifurcation in middle third of body, 311–355 (339), or 37.1–39.5% of total body length, from anterior extremity. Anterior caeca equal to subequal in length, much shorter than posterior caeca; left anterior caecum 28–48 (34); right anterior caecum 30–41 (34); longer anterior caecum occupying 3.5–5.1% of total body length. Posterior caeca equal to subequal in length, 2.9–5.5 (4.2) times longer than anterior; left posterior caecum 131–153 (141); right posterior caecum 127–142 (134); longer posterior caecum occupying 14.6–17.6% of total body length. Total caecal length 178–192 (183), occupying 20.0–21.2% of body length.

Testis single, roughly rectangular, with margins irregularly lobed, immediately posterior to posterior ends of posterior caeca, extends laterally beyond lateral nerve cords and posteriorly to anterior margin of ovary, 127–153 × 77–95 (138 × 84), occupying 14.3–16.7% of total body length; post-testicular space 264–310 (287), or 31.3–33.1% of body length. Vas deferens originates medially from posterior margin of testis, passing ovary and uterus ventrally, widening posteriorly in one specimen, entering cirrus-sac dorso-anteriorly. External seminal vesicle absent. Cirrus-sac retort-shaped, rounded anteriorly, dramatically narrowed posteriorly; anterior rounded portion 42–63 × 24–37 (51 × 31), contains seminal vesicle and pars prostatica; posterior narrow portion 80–98 (86) long, notably thickened at marginal genital pore, contains ejaculatory duct (un-everted cirrus; everted cirrus not observed), 4–7 (5) wide at midpoint, 6–9 (7) wide at marginal thickening. Seminal vesicle round to ovoid, 23–43 × 25–30 (35 × 28), restricted to anterior, rounded portion of cirrus-sac, joining coiled pars prostatica; prostatic cells not observed. Ejaculatory duct long. Male genital pore on sinistral margin at subtle marginal notch, 70–79 (74), or 8.1–8.7% of body length, from posterior extremity.

Ovary roughly rectangular or wedge-shaped, medial, with margins irregularly lobed, immediately posterior to testis, extending laterally beyond lateral nerve cords, 32–40 × 85–102 (37 × 94); post-ovarian space 241–284 (264), or 28.7–30.3% of total body length. Oviduct originates from posterior margin of ovary, passes posterio-dorsally to vitelline duct and dextro-lateral to ascending portion of uterus, posteriorly curving sinistrally to meet oötype, heavily distended with sperm. Oötype posterior to rest of genitalia, medial, surrounded by Mehlis’ gland, 75–94 (86) from posterior extremity. Uterus weakly convoluted, passing anteriorly between oviduct and dextral side of cirrus-sac, ventrally overlapping posterior margin of ovary, then passing posteriorly, sinistral to cirrus-sac, to female genital pore; distal portion of uterus often forming egg reservoir, creating distinct marginal bulge; egg reservoir sometimes distorting position of cirrus-sac. Female genital pore dorsal, sinistro-submedial, separate from and anterior to male pore, just posterior to level of constriction dividing anterior and posterior portions of cirrus-sac, 23–30 (26) from sinistral margin, 127–146 (138) from posterior extremity. Eggs *in utero* ovoid to subspherical, very thin-shelled, anoperculate, 25–28 × 16–22 (27 × 19). Vitellarium follicular, distributed from just posterior to dorsal nerve commissure to anterior margin of ovary, laterally exceeding nerve cords, largely confluent anterior to testis, interrupted partially by ends of caeca and oesophageal gland dorsally, interrupted partially by testis ventrally and dorsally. Vitelline duct passes ovary ventrally, passing posterio-dextrally to oötype, ventrally overlaps oviduct, posteriorly curving sinistrally to meet oötype.

Excretory vesicle small, pyriform; paired collecting ducts not traceable. Excretory pore at apex of terminal notch.

#### Remarks

Infections of *H. coronatus* were found in two of nine *S. diadema*; eggs were lodged in the gills of both infected hosts, and four adult worms (one worm in each of the four infected body sites) in one of the two. Sequence data for this species were derived from eggs in gill tissue.

### *Holocentricola* sp. A

*Host*: *Sargocentron rubrum* (Forsskål), Red squirrelfish (Holocentriformes: Holocentridae).

*Locality*: Off Heron Island (23° 27′ S, 151° 55′ E), southern Great Barrier Reef, Australia.

*Site in host*: Branchial arteries, vessels of liver, wash of head split.

*Prevalence*: 3 of 17 Heron Island (adult worms in three); 0 of 2 Lizard Island.

*Intensity*: 1 worm per fish, when adult worms were detected.

*Voucher material*: Two voucher specimens (QM G239129–30), both hologenophores.

*Representative DNA sequences*: Partial *cox*1 mtDNA, three identical sequences (one submitted to GenBank, OK421329); ITS2 rDNA, one sequence (submitted to GenBank, OK422502); partial 28S rDNA, one sequence (submitted to GenBank, OK422505).

#### Remarks

This putative, undescribed species was found co-infecting, with *H. rufus*, three individuals of *S. rubrum* at Heron Island; only three specimens were collected, and just two hologenophores were available for morphological analysis. This species is genetically distinct from but sister to *H. rufus* in all phylogenetic analyses; despite the shared host and close phylogenetic affinity, the two species are clearly distinct morphologically. Specimens of *Holocentricola* sp. A are much smaller than those representing *H. rufus*, with the two hologenophores being narrower (107–130 *vs.* 142–222), having a shorter testis length (70 *vs.* 100–233), post-testicular space (203–210 *vs.* 250–351), post-ovarian space (153–181 *vs.* 216–301), post-oötype space (55–74 *vs.* 81–138), post-male genital pore space (55–56 *vs.* 66–96), and post-female genital pore space (55–74 *vs.* 81–138). Specimens of *Holocentricola* sp. A also have fewer spines per row than *H. rufus* (7 *vs.* 8–9), smaller spines (6 long *vs.* 7–8 long) and shorter spine rows (8–11 wide *vs.* 11–22). The two hologenophore slides have been lodged as voucher specimens in the QM in the hope that future collecting in the region will enable the description of this species.

### Molecular results

**Figure 4:**
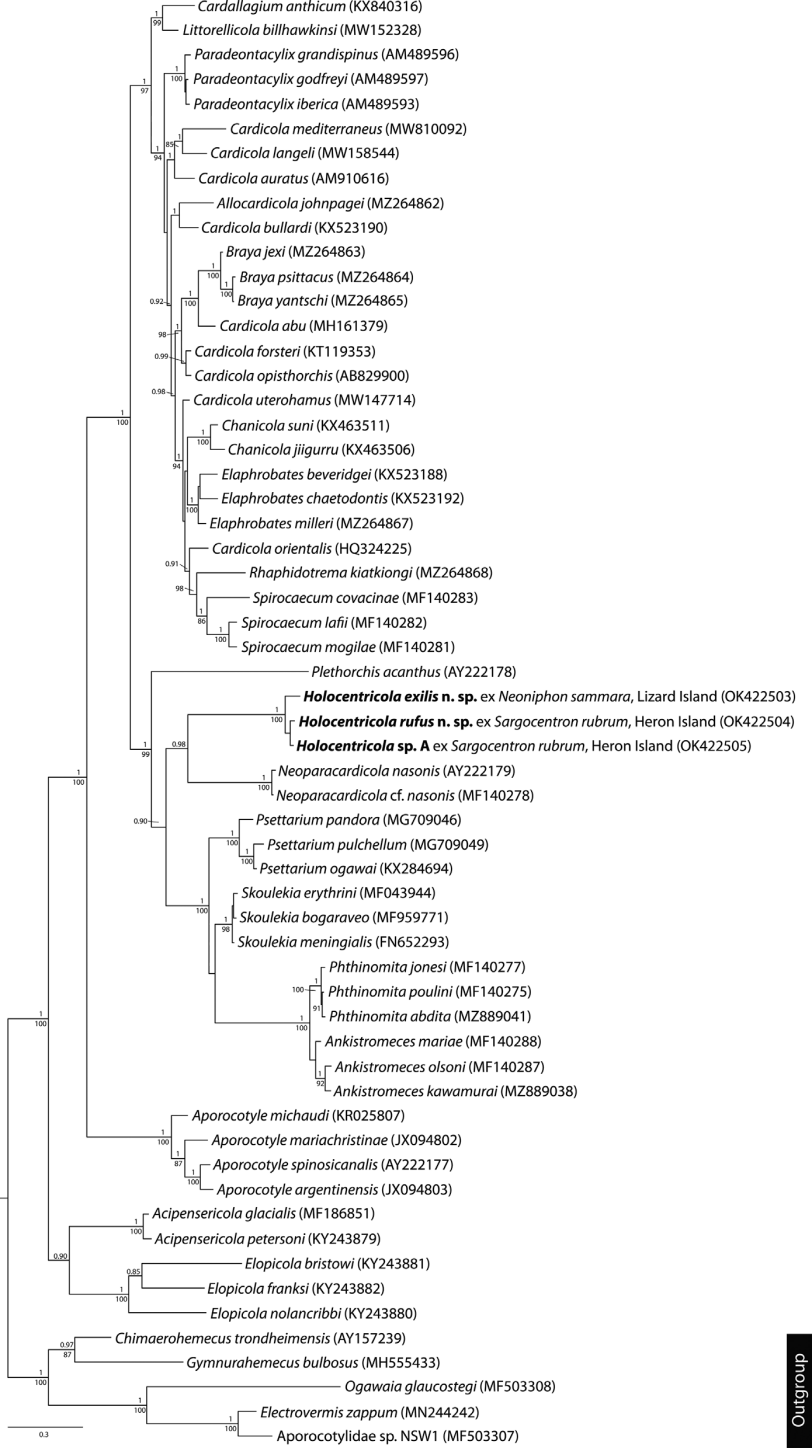
Relationships between species of *Holocentricola* and other members of the Aporocotylidae based on phylogenetic analysis of the 28S dataset. Bayesian inference posterior probabilities values are shown above the nodes and maximum likelihood bootstrap support shown below; values of <85 and <0.85 not shown. The scale-bar indicates expected number of substitutions per site.

*cox*1 and ITS2 data were generated for all four putative *Holocentricola* species, and 28S data for three species; genomic DNA of *H. coronatus* was derived from eggs lodged in gill filaments and thus the amplified 28S sequence was contaminated by host DNA. The four putative species of *Holocentricola* are clearly genetically distinct based on *cox*1 mtDNA and ITS2 rDNA data, differing at 34–61 base positions in the *cox*1 analysis (Fig. 1, Table 2) and 5–13 base positions in the ITS2 analysis. *cox*1 sequence data for *H. rufus* (the species for which the most replicate sequences were generated) demonstrated intra-specific variation at 0–5 base positions; no variation was found for three sequences of each of *H. exilis* and *Holocentricola* sp. A. Bayesian inference and maximum likelihood analyses of the 28S dataset resulted in identical phylograms (Fig. 4) in which species of *Holocentricola* form a strongly supported clade sister to that of *Neoparacardicola nasonis* Yamaguti, 1970 + *N*. cf. *nasonis* from *Naso unicornis* (Forsskål) (Acanthuridae) from the Great Barrier Reef. This clade is sister to a clade comprising species of *Ankistromeces*, *Phthinomita*, *Psettarium* and *Skoulekia* Alama-Bermejo, Montero, Raga & Holzer, 2011.

**Table 2:** Total pairwise *cox*1 differences between species of *Holocentricola*, with number of differences below the diagonal and *p*-distances above.

	1	2	3	4	5	6	7	8	9	10
1. *Holocentricola rufus* n. sp. (OK421322)		0.002	0.002	0.006	0.004	0.011	0.004	0.127	0.089	0.074
2. *Holocentricola rufus* n. sp. (OK421323)	1		0.004	0.004	0.006	0.008	0.002	0.129	0.091	0.076
3. *Holocentricola rufus* n. sp. (OK421325)	1	2		0.004	0.002	0.008	0.002	0.124	0.086	0.072
4. *Holocentricola rufus* n. sp. (OK421324)	3	2	2		0.006	0.008	0.002	0.127	0.089	0.074
5. *Holocentricola rufus* n. sp. (OK421326)	2	3	1	3		0.011	0.004	0.127	0.089	0.074
6. *Holocentricola rufus* n. sp. (OK421327)	5	4	4	4	5		0.006	0.129	0.091	0.072
7. *Holocentricola rufus* n. sp. (OK421328)	2	1	1	1	2	3		0.127	0.089	0.074
8. *Holocentricola exilis* n. sp. (OK421321)	60	61	59	60	60	61	60		0.118	0.108
9. *Holocentricola coronatus* n. sp. (OK421320)	42	43	41	42	42	43	42	56		0.097
10. *Holocentricola* sp. A (OK421329)	35	36	34	35	35	34	35	51	46	

## Discussion

*Holocentricola* can be immediately distinguished from all known aporocotylid genera in the structure of the cirrus-sac and the position of the male genital pore. All four putative species of the new genus possess a retort-shaped cirrus-sac, with rounded anterior section, a dramatically narrowed posterior section and a notable thickening/muscularisation at the marginal male genital pore. Although species of *Cardallagium* Yong, Cutmore, Jones, Gauthier & Cribb, 2017, *Cardicola* (sensu Yong et al. [73]), *Chanicola* Yong, Cribb & Cutmore, 2021, *Elaphrobates* Bullard & Overstreet, 2003 (sensu Yong et al. [73]), *Electrovermis* Warren & Bullard, 2019, *Littorellicola* Bullard, 2010, *Plehniella* Szidat, 1951 and *Psettarium* have been reported to have a marginal male genital pore, none have a retort-shaped cirrus-sac that is rounded anteriorly and dramatically narrowed posteriorly. Additionally, none of these genera, or any other in the Aporocotylidae, have been described with a distinct thickening of the cirrus-sac at the genital pore. In addition to the shape of the cirrus-sac, species of *Holocentricola* are distinct from those of other aporocotylid genera in having the combination of: (i) a lanceolate body; (ii) caeca that form an X-shape, with posterior caeca longer than anterior caeca; (iii) a single, post-caecal testis that is not deeply lobed; (iv) a post-caecal, post-testis ovary that is not distinctly bi-lobed; and (v) a post-ovarian uterus. In general body shape, the possession of X-shaped caeca and a single testis, species of *Holocentricola* are similar to those of *Braya*, *Cardicola*, *Chanicola*, *Cruoricola* Herbert, Shaharom-Harrison & Overstreet, 1994, *Elaphrobates*, *Parasanguinicola* Herbert & Shaharom, 1995, *Pearsonellum*, *Primisanguis* Bullard, Williams & Bunkley-Williams, 2012, *Skoulekia*, and *Spirocaecum* Yong, Cribb & Cutmore, 2021. However, the possession of a testis that is entirely post-caecal (rather than entirely or partially intercaecal) differentiates species of *Holocentricola* from those of all but *Parasanguinicola*, and the possession of a male genital pore that is marginal (rather than dorso-submarginal) differentiates them from those of *Parasanguinicola*, as well as from those of *Braya*, *Cruoricola*, *Skoulekia*, and *Spirocaecum*. Further, the ovary of species of *Holocentricola* is oblong, roughly rectangular or wedge-shaped with shallow, irregular lobes, not distinctly bi-lobed (as in species of *Chanicola* and *Cruoricola*) or dendritic (like that of the sole species of *Parasanguinicola*), and is post-caecal, not partially or completely intercaecal as in species of *Chanicola*, *Elaphrobates* and *Primisanguis*. Additionally, the uterus in species of *Holocentricola* never extends anteriorly beyond the ovary (unlike that in species of *Pearsonellum* and *Skoulekia*) or posteriorly past the oötype (unlike that of the sole species of *Primisanguis*). Finally, the new genus is phylogenetically distinct from species of *Braya*, *Cardicola*, *Chanicola*, *Elaphrobates*, *Skoulekia*, and *Spirocaecum* in our 28S analyses, forming a well-supported clade sister to but distinct from species of *Neoparacardicola* Yamaguti, 1970.

The three new species of *Holocentricola* described here are immediately distinguishable by size and row structure of the marginal spines. Most notably, the number of spines per row for the majority of the body length (they do reduce in number close to the anterior and posterior extremities in all three species) are different for each of the three species (*H. coronatus* with 5–6 per row, *H. exilis* with 7, *H. rufus* with 8–9). Further, *H. coronatus* has smaller spines than *H. rufus* and *H. exilis* (6 long *vs.* 7–8 and 8–9 long, respectively), which are arranged in narrower rows (8–9 wide *vs.* 11–28 and 10–14 wide, respectively). Additionally, *H. coronatus* has spine rows that are noticeable spaced closer together in the first third of the body length (3 apart) than in the posterior two thirds (4–5 apart); *H. rufus* and *H. exilis* have spine rows that are evenly spaced along the entire body length.

In addition to the differences in spination, the three species have non-overlapping oesophagus lengths (311–349 for *H. coronatus*, 359–420 for *H. exilis* and 433–597 for *H. rufus*), and *H. rufus* has a longer pre-bifurcal length than *H. coronatus* (39.5–48.2% of total body length *vs.* 37.1–39.5%). *Holocentricola rufus* has longer anterior caeca than *H. exilis* and *H. coronatus* (longer anterior caecum 6.1–8.8% of total body length *vs.* 3.3–6.2% and 3.5–5.1%, respectively), *H. exilis* has longer posterior caeca than *H. rufus* and *H. coronatus* (longer posterior caecum 19.3–24.5% of total body length *vs.* 9.5–19.5% and 14.6–17.6%, respectively), and thus *H. exilis* accordingly has a greater posterior caeca length to anterior caeca length ratio than *H. rufus* (posterior caeca 3.4–8.5 times longer than anterior *vs.* 1.3–2.9 times) and a longer total caecum length than *H. coronatus* (22.3–28.6% of total body length *vs.* 20.0–21.2%). *Holocentricola exilis* has the male genital pore further from the posterior extremity than *H. rufus* and *H. coronatus* (8.9–10.6% of body length from posterior extremity *vs.* 6.5–8.3% and 8.1–8.7%, respectively). Finally, *Holocentricola coronatus* is smaller than *H. rufus* and *H. exilis* (839–937 long *vs.* 976–1290 and 961–1232, respectively) and has a shorter post-ovarian space (28.7–30.3% of total body length *vs.* 21.4–24.3% and 21.1–25.4%, respectively), but has a longer post-testicular space (31.3–33.1% of total body length *vs.* 25.2–29.2% and 25.7–30.1%, respectively).

A notable feature in some specimens of all four species of *Holocentricola* was an expanded and heavily gravid distal portion of uterus, which we interpret as an egg reservoir; this reservoir was large enough to create a distinct sinistro-marginal bulge in egg-laden specimens. We infer that this structure relates to the egg laying habits of *Holocentricola* species. Examination of the gills under a compound microscope revealed that *Holocentricola* eggs were never randomly distributed across all or many of the filaments, rather they were always concentrated in clusters at the tip of just a small numbers of gill filaments; in the case of *H. exilis*, eggs were usually only present in one or two filaments of the entire gill structure. These egg clusters were large enough to be clearly visible during gross examination of the gills under a dissecting microscope and only a few individual eggs were found outside of these clusters. This is strikingly different from the seemingly random distribution of eggs across the gills reported for species of other aporocotylid genera (e.g. [8, 41, 49, 71]). We infer that gravid *Holocentricola* worms are highly mobile in the circulatory system and amass eggs in the reservoir, and, when laden, insert their posterior end into or enter a filament to lay. This interpretation is supported by the presence of the adults in sites throughout the circulatory system (the heart, branchial arteries, vessels of liver, head and body) and the rarity of eggs in the heart tissue of infected holocentrids; of the 81 holocentrids examined during this study just a single *S. rubrum* had a few eggs lodged in tissues of the ventricle. Eggs are routinely found lodged in the heart tissues (primarily the those of the ventricle) in fishes with a current or recent blood fluke infection (e.g. [52, 59, 68]); their absence in holocentrids suggests that *Holocentricola* eggs do not traverse the circulatory system passively. As far as we can determine, this pattern of confinement of the eggs to just a single or a few filament tips has not been reported for any other aporocotylids.

We suspect that the paucity of blood fluke reports for holocentrids prior to this study relates to a lack of examination of this family of fishes, rather than an absence of species infecting them. The semi-cryptic nature of holocentrids means they are not commonly seen and seldom collected. As part of the extensive blood fluke sampling during the PhD of Nolan [32–38] over 1200 individual fishes were examined from the Great Barrier Reef; these 1200 fishes included just a single holocentrid, a *N. sammara* from off Heron Island [31]. During our own long-term collection program, we have examined over 19,000 individuals of 960 marine teleost species, but just 14 of these species are holocentrids; of these 14 species we have examined just six for blood flukes (those in this study), and adequate numbers (at least 30 individuals, following Cribb et al. [11]) for just one (*N. sammara*). Given our findings of four blood fluke species in a small number of holocentrid species examined from just two locations, we predict that a more thorough survey of holocentrids in Australia (of which there are 34 species; Bray [4]), and elsewhere globally (a further 54 species; Froese and Pauly [18]), will reveal a rich aporocotylid fauna.

## Conclusions

This is the first report of aporocotylids from fishes of the family Holocentridae and the order Holocentriformes. The three new species described here (*Holocentricola coronatus*, *Holocentricola exilis* and *Holocentricola rufus*) are morphologically and genetically distinct from each other and from all other known aporocotylids. We predict that further examination of holocentrids will result in the collection of additional species of *Holocentricola*.

## Conflict of interest

The authors declare that they have no conflict of interest.
